# Growth Culture and Public Hospital Performance: The Mediating Effect of Job Satisfaction and Person–Organization Fit

**DOI:** 10.3390/ijerph191912185

**Published:** 2022-09-26

**Authors:** Change Xiong, Tong Hu, Ying Xia, Jing Cheng, Xiao Chen

**Affiliations:** 1 School of Public Health, Medical College, Wuhan University of Science and Technology, Wuhan 430065, China; 2 Department of Nursing, Medical College, Wuhan University of Science and Technology, Wuhan 430065, China; 3 School of Basic Medical Science, Hubei University of Science and Technology, Xianning 437100, China

**Keywords:** organizational culture, person–organization fit, job satisfaction, organizational performance, public hospital

## Abstract

This study investigates and interprets the role of growth organizational culture (GOC), person–organization fit (POF) and job satisfaction (JS) in predicting hospital performance (HP). This research adopted a quantitative methodology using data collected from 513 respondents in three cities across China. Smart-PLS 3 was used to evaluate the measurement model and structural model. Growth organizational culture has a significant positive impact on hospital performance (β = 0.191, *p* < 0.001). Person–organization fit (54.74%) and job satisfaction (29.26%) have partial mediating effects between growth culture and hospital performance. The research revealed that the GOC, POF, and JS play a substantial role in promoting HP. All the direct relationships were positive and significant. The findings suggest that establishing a growth culture environment for physicians is an effective strategy to improve physicians’ job satisfaction and person–organization fit. This strategy provides a new path to improve the hospitals performance through promoting organizational culture. Future studies should test the findings in an interventional design.

## 1. Introduction

Organizational culture refers to the values and beliefs that typify and prescribe norms of behavior in an organization. Although invisible and informal, it is an important force for managing organizations. A growth culture emphasizes innovation, creativity, openness and collaboration, while a conservative culture emphasizes authoritative leadership, guanxi and appearance [1]. Different types of organizational culture have different effects on organizational performance. This effect can be positive, negative or without effect at all. Organizations must increasingly maintain a culture to gain a competitive advantage. Research found that innovative culture has a significant positive impact on knowledge transfer performance, but whether supportive culture has an impact on knowledge transfer performance has not been confirmed [2,3]. One study showed innovative culture and supportive culture has a positive impact on organizational innovation performance, while bureaucratic culture has no significant effect on organizational innovation performance [4]. Several studies found the effects of various culture dimensions on performance [5,6,7]. A study found that organization culture flexibility is positively correlated with organizational growth performance, while the stability of organizational culture is negatively correlated. The more the organizational culture focuses on the outside, the higher the organizational performance, and the more the organization focuses on the inside, the lower the performance [8]. In China, more and more medical institutions are forming new consortiums, such as medical consortiums, medical communities, specialist alliances, internet hospitals, etc. Culture integration is a fact that cannot be ignored, and the new partnership needs to establish a more open and inclusive cultural atmosphere in order to promote better cooperation between institutions.

In recent years, organization culture, alongside structural reforms in order to improve healthcare performance, has been emphasized. Evidence from the research literature for a link between the culture of an organization and the quality and safety of the care that it provides is sparse. The link of organizational culture as related to hospital organization is weak from the research literature. Despite some evidence support establishing the links between culture and performance, however, much less is known regarding the mechanisms through which culture influences performance especially in healthcare organizations.

The purpose of our study, therefore, is to investigate and interpret the role of growth organizational culture (GOC), person–organization fit (POF), and job satisfaction (JS) in predicting hospital performance (HP) and when an organizational growth culture can effectively influence individual and organization value fit and increase job satisfaction. If physicians agree strongly about the direction and goals of hospitals, they continually renew efforts to achieve organizational goals. Therefore, our prediction is that the relationship between organizational growth culture and hospital performance can be partially explained through person–organization fit and job satisfaction.

This study expands the scope of previous study, exploring the relationship of growth organizational culture and hospital performance in hospital setting and discusses the moderating roles of job satisfaction and person–organization fit. We hypothesized that as growth organizational culture improved, hospital performance would improve.

### Literature Review and Hypothesis Development

**Hypothesis** **1** **(H1).** *Growth organization culture positively and significantly influences hospital performance*.

Much evidence indicates that organizational culture can promote organizational performance [9,10]. Hospital safety culture has been studied most and can influence medical quality [11]. Improved safety culture is associated with decreases in patient harm and hospital mortality across a hospital system [12], but safety culture is more about the norms and constraints of medical staff behavior. In the changing hospital system reforms, a growth culture which is more open, inclusive and encourages innovation is needed. The direct impact of organizational culture on organizational performance has always been focus of researchers, but there are still many uncertainties in the relationship between organizational culture and organizational performance. However, when an organization wants to make changes, the organizational culture that has been formed may hinder organizational performance [13]. In medical systems in China, hospitals have formed medical complexes in the form of urban hospital groups, specialty alliances and county medical communities. However, there are challenges in cultural integration.

The influence of organizational culture on organizational performance cannot be ignored in a multicultural workplace [14]. In the context of the establishment of various medical union in China, the former medical institutions have their own organization cultures, and the combination of institutions makes hospitals become multicultural workplaces.

**Hypothesis** **2** **(H2).** *Growth organization culture positively and significantly influences person–organization fit*.

Growth culture provides employees with more growth and development opportunities, so that employees can be more grateful to the organization, more strongly identify with the values and goals of the organization, effectively mobilize their enthusiasm and initiative, motivate employees to develop their own potential and improve work involvement. Growth culture is perceived as a competitive advantage for every organization for better selection and integration if the workforce is culturally diverse. It brings numerous advantages on the personal level and creates a safe climate in which physicians are inspired, supported and motivated [15]. Knowledge sharing culture can promote the relationship between the organization and the individual [16].

A harmonious organizational culture helps to form a good organizational atmosphere in which people’s behaviors are consistent with the values of the organization as far as possible. People’s values, behavior habits, personality characteristics, organizational culture, values, norms and so on constitute a good organizational cultural environment.

**Hypothesis** **3** **(H3).** *Growth organization culture positively and significantly influences job satisfaction*.

Organizational culture imposes significant impacts on staff and patient outcomes. In the US, management interventions targeting organizational culture have been proved to result in reductions in medical errors in hospitals [17]. A study conducted in Ethiopia showed that organizational culture is associated with staff satisfaction in primary hospitals [18]. Similarly, a significant correlation between organizational culture and quality of work life in hospital nurses was found in Korea [19].

Cameron and Quinn [20] examined organizational culture from four perspectives: clan, adhocracy, market and hierarchy. This theory is often used to judge the characteristics of organizational culture and explore the relationship between organizational performance. According to Hofstede, organizational culture is a complex of values and practices, and values are the core [21]. Hofstede’s theory is more appropriate at the level of national culture or society than organizations. Schein proposed three levels of organizational culture: artifacts, espoused beliefs and values and underlying assumptions [22]. Schein’s theory is often applied to the external adaptation and internal integration of organizations. These classical organizational culture theories guide organizations to provide more efficient organizational climates. Job satisfaction is very sensitive to the variations in organizational culture [18]. A perception of a highly people-oriented culture, for example, was found to be associated with lower workload, lower job strain, higher job satisfaction and lower intention to leave in hospital nurses in the US [23]. Teamwork and a clan culture can help boost staff satisfaction in the healthcare setting [18]. A study in Greece found the most important factor in doctors’ job satisfaction appears to be the nature of their job and the high levels of autonomy that they have [24].

Organizational culture can reduce employee stress in the workplace, and HR practitioners should match the types of organizational culture and employee competency situationally to reduce employee stress. Organizations desiring professional competency for their employees should build a clan organizational culture. While organizations should encourage a market organizational culture for their employees who possess result-oriented competency [25].

**Hypothesis** **4** **(H4).** *Person–organization fit positively and significantly influences hospital performance*.

Work performance of an employee is a reflection of actions for both personal and organizational purposes according to the person–organization value fit theory [26]. A good person–organization value fit is beneficial in maintaining staff wellbeing, which helps the passion for the work of employees [27,28]. A study showed that person–organization fit is associated with organizational citizenship behaviors (OCB) and the organizational commitments of staffs [29]. Its influence on organizational outcomes maybe much stronger than on work outcomes [29,30].

It is challenging to study the person–organization value fit of physicians, as physicians tend to serve as a consultant rather than an employee in the Chinese health context. Song and colleagues found that person–organization value fit is positively associated with medical workers’ engagement and negatively associated with turnover rate in public hospitals [31]. A study also found that person–organization value fit is positively correlated with self-efficacy of nurses [32]. We assume that the positive effects of person–organization value fit on physicians can improve hospital performance.

**Hypothesis** **5** **(H5).** *Job satisfaction positively and significantly influences hospital performance*.

Physicians’ job satisfaction refers to the general evaluation of their job [33]. Job satisfaction of staff is a quite important factor that influences organizational performance and therefore should be of major concern to organizations. Researchers have proposed several theories to interpret the link between job satisfaction and organizational performance, including the motivation theory and the wellbeing and productivity theory [34]. High job satisfaction means that physicians are happy at work and some evidence supports more job satisfaction resulting in better performance [35,36]. It is essential to know how staffs build organizational performance through their satisfaction and create competitive advantage in the workplace [37].

How public hospitals can best cultivate organization performance is becoming a challenging factor and they are adopting positive strategies for it. In this study, promoting job satisfaction of physicians is suggested to be one effective strategy to improve public hospital performance.

**Hypothesis** **6** **(H6).** *Person–organization value fit mediates the effect of growth organization culture on hospital performance*.

The influence of organizational culture on organizational performance is actually restricted by environmental factors. Person–organization value fit is one of important environmental factors and usually affects individuals’ psychology and behavior outcomes. Growth organizational culture is beneficial to the links between organization and employers. A study found person–organization fit significantly moderates the effects of job satisfaction on turnover intentions among employees at higher education institutions [38]. The mediating role of person–organization fit in the relationship between work environment and stress among social workers was verified [39]. A systematic review showed there was a positive association between value fit and staff outcomes in health settings [40]. Little literature has reported the cumulative effect of person–organization fit, job satisfaction and organization culture on hospital performance.

Combining matching theory and contingency management theory, this study believes that different organizational cultures have different effects on organizational performance, and the key is to match the internal and external environment of the organization. A culture well matched with the environment can mobilize the enthusiasm of employees, enhance the cohesion of employees and promote the improvement of organizational performance. However, a culture poorly matched with the environment will not be recognized by employees, will affect their organizational commitment and satisfaction and even hinder the improvement of organizational performance.

**Hypothesis** **7** **(H7).** *Job satisfaction mediates the effect of growth organization culture on hospital performance*.

Humans are the most precious resource in the organization, and the positivity of human resources in the organization directly determines the performance level of the organization. Therefore, the influence mechanism of organizational culture on organizational performance cannot be separated from the fundamental element of human resources. According to the viewpoint of modern psychology and behavior science, culture is an important factor affecting individual psychology and behavior, and the mechanism of the cultural field is realized through human beings. Therefore, it is a good choice to take employee psychology and behavior as the mediating variables. Different types of employee psychology and behavior can be selected according to different concerns.

A strong and balanced organization culture has a significant positive relationship with organizational performance, and this effect is mediated by employee attitudes [41]. However, most hospitals operate physicians’ job satisfaction as solely the responsibility of the individual physician and neglect organizational factors. Strengthening organizational culture is one of nine organizational strategies to promote engagement [42].

## 2. Materials and Methods

### 2.1. Sample and Procedure

A cross-sectional questionnaire survey of 513 hospital physicians was conducted in China. Implied informed consent was obtained from the participants prior to the survey. Ethics approval was granted by Hubei University of Science and Technology.

The survey was conducted in Zhejiang, Henan and Qinghai, representing eastern developed, central developing and western under-developed regions of China, respectively. The three provinces have a total of about 170 million inhabitants, accounting for 12.0% of China’s entire population size [43]. Hospital resources in China are heavily concentrated in large metropolitan areas. Across the three capital cities of the participating provinces, there are 65 tertiary public hospitals. In this study, two tertiary public hospitals from the capital city of each participating province were conveniently selected. All medical doctors employed by the participating hospitals were eligible for the study.

### 2.2. Data Collection

Data were collected over the period from October 2016 to August 2019. Four investigators were recruited from Hubei University of Science and Technology. They were trained to conduct the survey through face-to-face interviews. On the day of their visit, the medical doctors working in the hospital were approached and invited to participate in the survey. In total, 720 medical doctors were invited and 513 (71%) accepted and completed the survey. The sample represented 16.57% of the medical doctors (*n* = 3096) working in the six participating hospitals.

The interviews took place in the preferred office space of the study participants. Prior to each interview, the participant was asked to read the information statement and provide implied informed consent. The survey was voluntary and anonymous. Each interview took about 20 min. The investigators did not have any working or servicing relationship with the study participants. The respondents were allowed to withdraw at any stage of the interviews.

### 2.3. Measures

This survey comprised four scales (Appendix A): (1) growth organizational culture, (2) person–organization fit, (3) job satisfaction and (4) hospital performance.

#### 2.3.1. Growth Organizational Culture

Growth organizational culture was measured by a validated scale developed by Zheng and colleagues, which was adapted to the context of the Chinese culture [44]. Zheng defined organizational culture as “the normative and internalized beliefs of the members of an organization that can guide the behavior and management style of the entire organization” [45]. The scale’s 10 descriptive items measured cultural tendency towards growth approaches in hospital development. A growth culture emphasizes innovation, creativity, openness and collaboration [1]. Example questions include “managers respect individual wishes”. Respondents were asked to rate each item on a five-point Likert scale ranging from 1 “very low” to 5 “very high” according to their experience. A summed mean score (ranging from 1 to 5) was calculated, with a higher score indicating a higher tendency. The Cronbach’s α coefficient was greater than 0.9 for the growth scale.

#### 2.3.2. Person–Organization Fit

Person–organization fit was defined as perceived value congruence between an individual and their organization [46]. This study adopted the Chinese version of the person–organization fit instrument developed by Cable and Judge, which has been validated [47]. Four items measured perceived fitness of individual values with those of the organization (hospital). The concept of value for individuals is inherently subjective [48]. Therefore, measuring perceived person–organization fit is important. In this study, respondents were asked to rate their perceived value fitness on a five-point Likert scale, ranging from 1 (not at all) to 5 (completely). A summed mean score was calculated (ranging from 1 to 5), with a higher score indicating higher person–organization fit. The Cronbach’s α coefficient of the person–organization fit scale was greater than 0.9 in this study.

#### 2.3.3. Job Satisfaction

Kalleberg [49] defined job satisfaction as “the affective attitudes of an employee toward her/his work after balancing various aspects of the work”. The job satisfaction scale developed by Tsui and colleagues [50] was adopted in this study, in line with Kalleberg’s definition. The scale has been validated [51], including its Chinese version [52]. Four items measured satisfaction of employees in the work itself, personal responsibility, colleagues, superiors and remuneration and promotion, respectively. Respondents were asked to rate their satisfaction on a five-point Likert scales, ranging from 1 (strongly disagree) to 5 (strongly agree). A summed mean score (ranging from 1 to 5) was calculated, with a higher score indicating higher job satisfaction. The Cronbach’s α coefficient of the job satisfaction scale was greater than 0.8 in this study.

#### 2.3.4. Hospital Performance

In this study, participants were asked to rank their hospital performance against their regional competitors on a quintile scale ranging from 1 (bottom 20%) to 5 (top 20%). Four items were adapted from the instrument developed by Tan and Litschert [52,53] measuring performance of the hospitals. This instrument has been validated in China [54]. An average summed score was calculated, with a higher score indicating higher hospital performance. This study adopted the Tan and Litschert instrument to evaluate hospital performance for two reasons. Firstly, this instrument emphasizes financial operations, which are closely associated with remuneration of employees in public hospitals in China. Secondly, this instrument adopts a self-rating approach. Tan and Litschert [53] suggest that employees are well positioned to compare the relative performance of their organization with its close competitors. Previous studies have proven that perceptive performance can serve as a useful alternative measures of objective performance [55]. Compared with objective performance data, self-rating can better reflect perceived individual contributions of an employee to the hospital. Hospital performance is a result of collective efforts and individuals are not necessarily able to contribute to all aspects of the hospital. Perceptive measurements build a natural connection between individual employees and hospital performance.

### 2.4. Statistical Analysis

Mean values and standard deviations (SD) of the measured constructs were calculated and compared between the respondents with different sociodemographic characteristics using student *t* tests or ANOVA. The SPSS version 26 was used to perform the tests.

**Figure 1:**
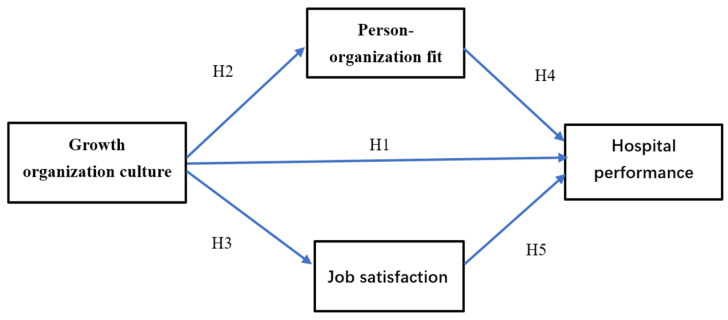
Conceptual model.

Partial least squares structural equation modeling (PLS-SEM) was performed to test the links between organizational culture, person–organization fit, job satisfaction, and hospital performance. PLS-SEM is a non-parametric method that does not have the same strict requirements on the distribution of data as the parametric method [56]. It also offers some features that are particularly suitable for the purpose of this study. PLS-SEM allows us to explore the mediating role of person–organization fit and job satisfaction simultaneously in the link between growth organizational culture and hospital performance (Figure 1).

We adopted a two-step approach to establish the structural model. The first step was to ensure sufficient reliability and validity of the measurements. The reliability of the measurements was assessed using the coefficients of Cronbach’s alpha (CA > 0.7), composite reliability (CR > 0.7) and average variance extracted (AVE > 0.5). The validity of the measurements was assessed using the Fornell–Lacker criterion on item loadings for convergent validity [55,57]. According to Hair et al. [58], the loading of each measurement item on its respective construct (latent variable) should be greater than 0.70. However, removal of any items with a lower than 0.70 load should be considered only if the deletion increases the composite reliability. We also tested the discriminant validity of the measured constructs: the square root of AVE of each construct should exceed its correlations with other constructs [55]. The second step involved assessment of data fitness into the proposed structural model as well as significance tests of path coefficients embedded in the model. Before the establishment of the structural model, collinearity across items within each measured construct was tested using the variance inflation factor (VIF) value, with a value below 5.0 being deemed acceptable [59]. Kock and Lynn argue that deleting items with high collinearity can improve modeling efficiency [60]. Bootstrapping with 5000 samples was used in this study to estimate the path coefficients as suggested by Hair et al. [58]. A standardized root mean residual (SRMR) of less than 0.08 was deemed acceptable fitness for data in the model [61].

## 3. Results

### 3.1. Descriptive Statistics

The majority of study participants were women (65.11%) and younger than 40 years (57.12%), had a postgraduate degree (65.89%), earned a monthly salary between ¥2000 and ¥5000 Yuan (63.94%), had less than 6 years of work experience (56.53%) and held a junior professional title (59.06%).

The study participants reported an average score of 3.47 for growth organizational culture (SD = 0.74), 3.30 for person–organization fit (SD = 0.80), 3.33 for job satisfaction (SD = 0.70) and 3.04 for hospital performance (SD = 0.97).

The female respondents had lower scores in job satisfaction (*p* = 0.004), hospital performance (*p* < 0.001) and person–organization fit (*p* < 0.001) than their male counterparts. Those with a lower income reported lower job satisfaction (*p* < 0.001), poorer hospital performance (*p* < 0.001) and lower person–organization fit (*p* < 0.001). Higher income was associated higher levels of growth culture (*p* = 0.021). Similarly, the respondents with less work experience perceived lower growth culture (*p* = 0.020) and reported lower job satisfaction (*p* = 0.002), lower person–organization fit (*p* = 0.001) and higher hospital performance (*p* < 0.001) than their more experienced colleagues (Table 1).

### 3.2. Measurements Model

Items of scales loadings above 0.7 indicate the construct explains more than 50 per cent of the indicator’s variance [58]. The items produced acceptable reliability, with the composite reliability coefficients and Cronbach’s α far exceeding the recommended value of 0.7 [58] (Table 2). While Cronbach’s alpha may be too conservative, the composite reliability may be too liberal and the construct’s true reliability is typically viewed as within these two extreme values. As an alternative, Dijkstra and Henseler proposed ρA as an approximately exact measure of construct reliability, which usually lies between Cronbach’s α and the composite reliability [61].

Convergent validity was evaluated using the average variance extracted (AVE) [58]. AVE of every scale exceeds the minimum threshold value of 0.5 in this study.

The discriminant validity of the measured constructs was analyzed through Fornell and Larcker (Table 3), but Henseler et al. [62] proposed the heterotrait/monotrait ratio of correlations (HTMT) replaced Fornell and Larcker criterion when the indicator loadings on a construct differ only slightly. Table 4 shows that all constructs had acceptable discriminant validity using HTMT criterion and HTMT values not exceeding the value of 0.85 [63].

### 3.3. Structural Model

**Figure 2:**
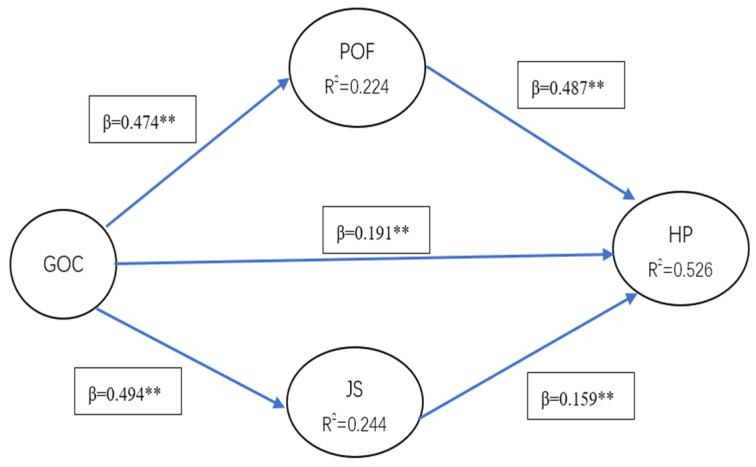
Structural model ** *p* < 0.01.

The structural model analysis was performed using standardized paths and path analysis are shown in Table 5 and Figure 2.

The structural model’s predictive ability was evaluated using bootstrapping and blindfolding procedures. Path coefficients and R^2^ (0.526) evaluate the degree of significance of model, and Q^2^ evaluates predictive relevance [64]. The results of blindfolding algorithm show high Q^2^ (0.397) effect size for hospital performance. This value depicts a high predictive relevance as Q^2^ is larger than zero [58]. The bootstrapping procedure calculates SRMR value is 0.061, which is within the acceptable value of SRMR as smaller than 0.08. Furthermore, to demonstrate the predictive relevance, the PLSpredict algorithm is used to predict the PLS model’s performance for the Manifest Variable (MV) and Latent Variable (LV) [64,65]. The PLSpredict algorithms involve cross-validated casewise and average-case point predictions (Table 6). MAPE (mean absolute percentage error), RMSE (root mean square error) and MAE (mean absolute error). The analysis uses 10-fold and 10 repetitions to perform the PLSpredict estimation. Linear model (LM) predictions such as naïve benchmarks to measure the predictive quality of PLS path model estimations. If PLS-SEM analysis (compared to the LM) yields higher prediction errors in terms of RMSE (or MAE, MAPE) for the minority, it showed medium predictive power [58].

#### Mediation Analysis

The results reveal that growth organizational culture has strong direct effect on hospital performance. On the other hand, the indirect impact of growth organizational culture on hospital performance through person–organization fit and job satisfaction is also significant. Person–organization fit and job satisfaction were significant predictors of hospital performance, which absorbed 54.74% (POF) and 29.26% (JS) of the effect of growth organizational culture on hospital performance as measured by variance accounted for (VAF). Consequently, person–organization fit and job satisfaction partially mediate the relationship between growth organizational culture and hospital performance. Hypotheses H6 and H7 are accepted (Table 7).

## 4. Discussion

### 4.1. Main Findings

High quality development has been advocated in the healthcare fields in China since 2021. Building new culture is one of five new tasks to promoting public hospitals performance. Hospital culture always emphasized regime and hierarchy as was influenced by Confucianism, especially in public hospitals, including what kind of culture needs to be explored constantly in public service industries. However, several studies have been conducted on organizational culture, but the factors that influence were still unexplored in public hospitals. This study found HP is influenced by the GOC, JS and POF. Additionally, the R^2^ value confirms that GOC, POF and JS are good predictors of HP. Previous study also showed HP was influenced by organizational culture [66]. The standardized path coefficient GOC-HP in the structural equation models indicates that the GOC positively and significantly influences HP. The value shows that because of GOC, the hospitals evolve higher performance. The effect of organizational culture is based on the personal well-being and value match. The findings are consistent with other researches, as if physicians feel organizational culture can promote their more well-being, they will more positively work for organizational development, so the reason for the rise in hospital performance was growth culture.

Although some studies have focused on the effect of job satisfaction or person–organization fit alone on HP, the cumulative effect of JS, POF and GOC on HP has not been fully elucidated. Our results may reduce this literature gap. Previous studies suggested that when the growth organizational culture score is high, physicians experience an increased level of job satisfaction and person–organization fit [11,18]. Furthermore, the path coefficients GOC-POF and GOC-JS indicate that JS and POF are significantly influenced by GOC. The values show that GOC increased the JS and POF among the physicians in public hospitals. GOC is more open, innovative, inclusive and sensitive to personal feelings than traditional cultures. Hence, GOC makes physicians more satisfied with their working environment, atmosphere and personal growth. On the other hand, the path coefficients JS-HP and POF-HP show that JS and POF influence HP positively and significantly, and HP is more influenced by POF than JS. According to the literature, when POF is high, physicians are more willing to work toward organizational goals as they perceive that organizations focus on individual values and encourage individuals’ behaviors [40,67]. Similarly, JS affects hospital performance [24,68], and the higher the JS level is, the stronger the internal motivation of physicians’ work is, which promotes organizational performance. High level matches not only affect physicians’ feelings but also work behavior. Physicians with higher POF are more satisfied with the organizational environment and more willing to work actively. This implies that POF is more impactful than JS in hospital performance. Hence, the findings support the literature that JS and POF substantially affect the hospital performance. The GOC has positively impacted HP through JS and POF in the presence of JS and POF. Thus, JS and POF partially mediate the effect of GOC and HP. Hence, the GOC directly and indirectly affects HP.

### 4.2. Theoretical Implication

This study contributes to the literature in several ways. Even though some of the most prominent research in organizational performance has repeatedly demonstrated the influence of culture factors on performance, only a limited number of studies have explored growth culture impact on hospital performance. Firstly, several studies have been conducted on organizational culture as an independent variable [6,18]; there were few studies on how growth organizational culture (GOC) affects organizational performance [69,70], and this study contributes in this regard. This study highlights the GOC that influences organizational performance. Now, GOC is considered the important predictor of hospital performance. Second, the current research explores the mediating role of JS and POF between GOC and HP. However, these variables were infrequently used as mediators. Hence, this study will contribute to the body of knowledge by exploring the rarely discussed direct and mediating relationship between GOC and HP. Third, the research on public hospital performance settings is still scarce due to its novelty; this study will also contribute in this regard.

### 4.3. Practical Implication

The study’s findings have practical implications for health staff, hospital administrators and health policy makers. It has been empirically that GOC leads toward JS and POF which is an important outcome upon the fact that if the hospital creates an interaction environment for the physicians it brings JS and better organizational identification among physicians. Thus, GOC has double benefits for the hospitals and physician involvement in rationalized organizational performance. Hospitals can ensure the maximum performance by being involved in positive organizational culture atmosphere.

Secondly, this study examines the effect of the GOC on hospital performance, which could potentially pave the way for a bright future for the public hospital performance in China. As promoting growth culture is likely to become a management process or rule in the future, these findings can assist hospital managers and health departments in focusing the role growth culture in hospital performance.

## 5. Conclusions

The research revealed that the GOC, POF and JS play a substantial role in promoting HP. Based on empirical findings of this, GOC has a link with HP. Thus, it can be recommended that public hospitals emphasize open, inclusive and innovative climates while regulating physician behavior and growth culture to become a source to promote hospitals performance. Later, it may be empirically found that GOC is correlated with JS and POF, which is also an important outcome and stresses upon the fact that if public hospitals create a growth organizational culture environment for the physicians it may bring job satisfaction and person–organization value fit among physicians. Moreover, it has been found that JS and POF play mediating roles between the relationships of GOC and HP. JS and POF carry out the effect of GOC, passing this to the HP of the public hospitals. Hence, the GOC is associate with work outcomes, it also is beneficial to performance for public hospital. Overall, the study’s findings corroborate the theory by conforming that GOC, JS and POF were beneficial to HP.

### Limitation and Future Research

This study has certain limitations. (1) It has a sample size of 513, which is a small sample concerning the population of the physicians. Future research should be conducted with a larger sample sizes and probability sampling to ensure rigor in the study. (2) Growth organizational culture is one type of the various types of culture, and more research is needed to find out other culture types how to effect organizational performance. (3) The variable endogeneity is not well solved, which affects the generalization of the conclusion. Future research should adopt many methods to solve the variable endogeneity. (4) R^2^ shows that HP is 52.6% influenced by GOC, JS and POF; thus, there are other influencing HP factors must be considered. (5) This study focused only on physicians in public hospitals. The recommendation is to target the other medical staff, such as nurses. (6) While this study is restricted to tertiary public hospitals in China, future study should be undertaken in different contexts, such as private hospitals, specialized hospitals, hospitals for traditional Chinese medicine and so on.

## Figures and Tables

**Table 1:** Growth organization culture, job satisfaction, person–organization fit and hospital performance reported by the study participants by sociodemographic characteristics.

Characteristics		*n*	%	Growth Organizational Culture	Job Satisfaction	Hospital Performance	Person–Organization Fit
Gender							
	Male	179	34.89	3.55 ± 0.69	3.45 ± 0.76	3.278 ± 0.99	3.45 ± 0.76
	Female	334	65.11	3.43 ± 0.76	3.27 ± 0.67	2.91 ± 0.94	3.27 ± 0.67
				*p* = 0.062	*p* = 0.004	*p* < 0.001	*p* < 0.001
Age (Years)							
	30–39	293	57.12	3.49 ± 0.71	3.38 ± 0.70	3.10 ± 0.90	3.34 ± 0.77
	40–49	153	29.82	3.44 ± 0.73	3.27 ± 0.65	2.99 ± 1.03	3.27 ± 0.65
	50–59	67	13.06	3.45 ± 0.88	3.25 ± 0.84	2.89 ± 1.09	3.25 ± 0.84
				*p* = 0.802	*p* = 0.166	*p* = 0.181	*p* = 0.982
Educational attainment							
	Bachelor’s degree	175	34.11	3.48 ± 0.76	3.30 ± 0.65	3.14 ± 0.83	3.27 ± 0.73
	Postgraduate degree	338	65.89	3.47 ± 0.73	3.35 ± 0.73	2.99 ± 1.03	3.31 ± 0.83
				*p* = 0.910	*p* = 0.410	*p* = 0.09	*p* = 0.598
Monthly income							
	2000–5000	328	63.94	3.41 ± 0.76	3.23 ± 0.70	2.88 ± 0.91	3.14 ± 0.81
	5001–10,000	141	27.48	3.55 ± 0.70	3.13 ± 0.99	3.79 ± 0.62	3.48 ± 0.73
	≥10,000	44	8.58	3.68 ± 0.63	3.96 ± 0.78	3.84 ± 0.63	3.87 ± 0.50
				*p* = 0.021	*p* < 0.001	*p* < 0.001	*p* < 0.001
Working experience (years)							
	1–5	290	56.53	3.54 ± 0.69	3.42 ± 0.70	3.20 ± 0.92	3.41 ± 0.70
	6–10	123	23.98	3.43 ± 0.73	3.28 ± 0.62	2.94 ± 0.99	3.28 ± 0.62
	11–20	100	19.49	3.31 ± 0.84	3.15 ± 0.76	2.71 ± 0.98	3.15 ± 0.76
				*p* = 0.020	*p* = 0.002	*p* < 0.001	*p* = 0.001
Professional title							
	Junior	303	59.06	3.47 ± 0.71	3.37 ± 0.68	3.10 ± 0.98	3.35 ± 0.80
	Intermediate	173	33.73	3.48 ± 0.73	3.32 ± 0.69	3.00 ± 0.92	3.23 ± 0.76
	Senior	37	7.21	3.47 ± 0.96	3.33 ± 0.70	2.70 ± 1.00	3.15 ± 0.90
				*p* = 0.981	*p* = 0.168	*p* = 0.048	*p* = 0.129

**Table 2:** Measurement model results.

Items	Loadings	Cronbach’s α	Rho_A	Composite Reliability	AVE
Job Satisfaction (JS)		0.817	0.830	0.880	0.649
JS-1	0.732				
JS-2	0.733				
JS-3	0.876				
JS-4	0.868				
Hospital Performance (HP)		0.898	0.898	0.929	0.765
HP-1	0.868				
HP-2	0.881				
HP-3	0.875				
HP-4	0.874				
Person–Organization Fit (POF)		0.863	0.873	0.907	0.711
POF-1	0.860				
POF-2	0.881				
POF-3	0.880				
POF-4	0.745				
Growth Organization Culture (GOC)		0.933	0.937	0.943	0.624
GOC-1	0.733				
GOC-2	0.797				
GOC-3	0.774				
GOC-4	0.814				
GOC-5	0.741				
GOC-6	0.820				
GOC-7	0.788				
GOC-8	0.816				
GOC-9	0.812				
GOC-10	0.799				

Notes: GOC = growth organizational culture; JS = job satisfaction; HP = hospital performance; POF = person–organization fit.

**Table 3:** Discriminant validity: Fornell–Larcker criterion.

Construct	Growth Organization Culture (GOC)	Hospital Performance (HP)	Job Satisfaction (JS)	Person–Organization Fit (POF)
GOC	0.790			
HP	0.500	0.875		
JS	0.494	0.597	0.805	
POF	0.474	0.689	0.706	0.843

Notes: GOC = growth organizational culture; JS = job satisfaction; HP = hospital performance; POF = person–organization fit.

**Table 4:** Discriminant validity of heterotrait/monotrait ratio (HTMT).

Construct	Growth Organization Culture (GOC)	Hospital Performance (HP)	Job Satisfaction (JS)	Person–Organization Fit (POF)
GOC				
HP	0.534			
JS	0.553	0.690		
POF	0.511	0.781	0.827	

Notes: GOC = growth organizational culture; JS = job satisfaction; HP = hospital performance; POF = person–organization fit.

**Table 5:** Structural relationship and hypothesis testing.

Path	Path Coefficient	t	*p*	f^2^	95% Confidence Intervals		Decision
GOC→HP	0.191	4.544	<0.01	0.056	0.106	0.271	H1 accepted
GOC→POF	0.474	13.181	<0.01	0.289	0.403	0.544	H2 accepted
GOC→JS	0.494	14.385	<0.01	0.322	0.426	0.561	H3 accepted
POF→HP	0.487	9.912	<0.01	0.240	0.387	0.579	H4 accepted
JS→HP	0.159	3.230	0.001	0.025	0.387	0.579	H5 accepted
R^2^ (HP)	0.526						
Q^2^ (HP)	0.397						

Notes: GOC = growth organizational culture; JS = job satisfaction; HP = hospital performance; POF = person–organization fit.

**Table 6:** PLS predict results.

	RMSE		MAE		MAPE		Q2	
	LM	PLS-SEM	LM	PLS-SEM	LM	PLS-EM	LM	PLS-SEM
HP-1	0.97	0.99	0.79	0.81	39.97	41.42	0.22	0.18
HP-2	0.99	0.99	0.80	0.79	36.90	36.79	0.16	0.16
HP-3	1.02	1.00	0.81	0.81	33.82	33.56	0.16	0.18
HP-4	0.97	0.98	0.77	0.78	34.08	34.90	0.24	0.22
JS-1	0.90	0.91	0.72	0.72	33.35	34.13	0.16	0.15
JS-2	0.90	0.89	0.72	0.73	27.39	27.73	0.12	0.13
JS-3	1.03	1.06	0.85	0.89	42.74	45.56	0.21	0.16
JS-4	0.93	0.93	0.75	0.76	32.75	33.29	0.18	0.17
HP-1	0.83	0.84	0.66	0.68	29.20	30.52	0.23	0.21
HP-2	0.84	0.85	0.66	0.67	27.52	28.21	0.15	0.13
HP-3	0.87	0.88	0.69	0.71	29.24	30.11	0.19	0.18
HP-4	0.83	0.82	0.65	0.64	24.23	24.03	0.07	0.09

**Table 7:** Mediation analysis.

Path	Direct Effect	Indirect Effect	Total Effect	Variation Accounted For (VAF)	T Value	95% Confidence Intervals		Decision
GOC→POF→HP	0.191	0.231	0.422	54.74%	8.054	0.178	0.291	H6 accepted
GOC→JS→HP	0.191	0.079	0.27	29.26%	3.126	0.033	0.131	H7 accepted

Notes: GOC =growth organizational culture; JS = job satisfaction; HP = hospital performance; POF = person–organization fit.

## Data Availability

The original contributions presented in the study are included in the article, and further inquiries can be directed to the corresponding authors.

## Appendix A

**Table A1:** Items of scales.

Variables	Indicators	Items
JS	JS-1	Satisfaction with promotion opportunity
	JS-2	Satisfaction with individual tasks
	JS-3	Satisfaction with remuneration
	JS-4	Overall rating
HP	HP-1	Hospital profits
	HP-2	Hospital revenue growth
	HP-3	The mental outlook of hospital staff
	HP-4	The hospital’s competitive position in the region
POF	POF-1	Personal value reflected in organizational value
	POF-2	Personal personality and organizational trait fit
	POF-3	Person–organization match
	POF-4	Person–colleague value match
GOC	GOC-1	Encourage innovation and invention
	GOC-2	Work as a team
	GOC-3	Respect individual wishes
	GOC-4	Pursue excellence
	GOC-5	Integrity in work style
	GOC-6	Committed to science and truth
	GOC-7	Pay attention to harmony
	GOC-8	Aggressive
	GOC-9	Equitable reward and punishment
	GOC-10	Respect institutional norms
